# Diagnostic and Therapeutic Challenges of Hepatoadrenal Syndrome in Advanced Cirrhosis: A Case Report and Literature Review

**DOI:** 10.7759/cureus.96489

**Published:** 2025-11-10

**Authors:** Abban Bashir, Idowu Olaogun

**Affiliations:** 1 Medicine, University Hospitals Bristol and Weston National Health Service (NHS) Foundation Trust, Bristol, GBR

**Keywords:** adrenal insufficiency, alcohol-related liver disease, endocrine, hepatology, relative adrenal insufficiency

## Abstract

Cirrhosis is a complex multisystem disorder with significant endocrine, metabolic, and circulatory consequences. One under-recognised complication is hepato-adrenal syndrome, or relative adrenal insufficiency (RAI), where adrenal dysfunction may contribute to haemodynamic instability. Diagnosing RAI in cirrhotic patients is challenging due to clinical overlap with other cirrhosis-related conditions, biochemical confounders such as hypoalbuminemia and reduced corticosteroid-binding globulin, and unclear interpretive thresholds for cortisol testing.

We report a case involving a 58-year-old man with alcohol-induced decompensated cirrhosis presenting with persistent hypotension, hyponatraemia, and hyperkalaemia. A short Synacthen test revealed a suboptimal cortisol response, prompting empiric corticosteroid therapy. However, salivary cortisol, representing a free, biologically active hormone, was within normal limits, casting doubt on the diagnosis of actual adrenal insufficiency. Despite prolonged steroid administration, the patient deteriorated and ultimately died from multiorgan failure.

This case highlights diagnostic limitations of current RAI evaluation strategies in cirrhosis, where reduced serum cortisol may not reliably reflect adrenal capacity and where dynamic testing lacks specificity in altered hepatic physiology. Additionally, it raises concern about the potential harms of empiric corticosteroid therapy, including receptor downregulation and iatrogenic complications.

A review of the literature supports cautious steroid use in critically ill cirrhotic patients, with treatment guided by objective clinical and biochemical responses. Until cirrhosis-specific diagnostic criteria for RAI are established, clinicians must integrate laboratory findings with clinical context, medication effects, and evolving haemodynamics. This case advocates for reevaluation of diagnostic algorithms and individualised treatment thresholds in end-stage liver disease.

## Introduction

Chronic liver disease remains a major global health burden, responsible for significant morbidity and mortality due to its systemic complications. Beyond progressive hepatic dysfunction, advanced cirrhosis is characterised by widespread circulatory (hyperdynamic vasodilation with renal hypoperfusion), immunologic (cirrhosis-associated immune dysfunction with high infection risk), and metabolic disturbances (e.g., ammonia production contributing to hepatic encephalopathy and renal sodium retention), which precipitate failure of distant organs. Well-recognised syndromes such as hepatorenal syndrome, hepatic encephalopathy, and spontaneous bacterial peritonitis exemplify how cirrhosis can provoke multiorgan failure through functional, rather than structural, mechanisms [1].

Hepato-adrenal syndrome, an evolving and relatively underrecognised manifestation of this phenomenon, refers to relative adrenal insufficiency, a state in which the adrenal glands fail to produce sufficient biologically active free cortisol to meet the metabolic demands of severe illness despite potentially normal total cortisol levels [2,3]. This distinction is crucial, as total serum cortisol can be misleading in cirrhosis due to reduced cortisol-binding globulin, hypoalbuminaemia, and altered hepatic metabolism, all of which compromise the reliability of conventional cortisol assays.

Clinically, RAI in cirrhosis is particularly challenging to identify because its clinical manifestations, including hypotension, electrolyte abnormalities, and reduced vascular tone, overlap with other cirrhosis-related syndromes and can mimic classical adrenal insufficiency, making recognition difficult and timely management difficult [3]. The lack of standardised diagnostic criteria, limitations of cortisol assays in cirrhosis, and uncertain benefit of corticosteroid therapy further complicate clinical decision-making. These diagnostic ambiguities highlight the need for careful clinical interpretation and consideration of alternative measures of adrenal function, such as salivary cortisol, which may better reflect the biologically active fraction in advanced liver disease.

Epidemiologically, RAI affects roughly one-quarter to one-third of hospitalised, non-critically ill patients with cirrhosis. Siramolpiwat et al. reported a prevalence of 30.4% and showed that affected patients had more advanced liver dysfunction by Child-Pugh and MELD scores [4]. Acevedo et al. have reported prevalence figures in the mid-20% range. In the setting of critical illness or severe sepsis, the proportion rises above 60%, indicating that disease severity and acute inflammatory stress amplify the likelihood of an inadequate adrenal response [5].

Clinically, RAI carries important prognostic implications. Short-term complications, including bacterial infection, severe sepsis, hepatorenal syndrome, and early death, occur more often in cirrhotic patients with impaired cortisol responses [5]. Long-term outcomes are also worse: one longitudinal study observed a one-year survival rate of 69.2% in patients with RAI compared with 95.0% in those with preserved adrenal function [6], corresponding to an approximate one-year mortality rate of 30.8% versus 5.0%. Pathophysiologically, low cholesterol substrate, pro-inflammatory cytokine-mediated suppression of the hypothalamic-pituitary-adrenal axis, and endotoxin exposure contribute to this blunted stress response [7].

Together, these findings highlight that recognising RAI is both epidemiologically relevant and clinically significant, as it identifies patients at greater risk of decompensation and reduced survival. The following report further explores the diagnostic uncertainty and therapeutic challenges associated with presumed relative adrenal insufficiency in advanced cirrhosis, focusing on how altered cortisol physiology and medication effects complicate its interpretation and management.

## Case presentation

**Figure 1:**
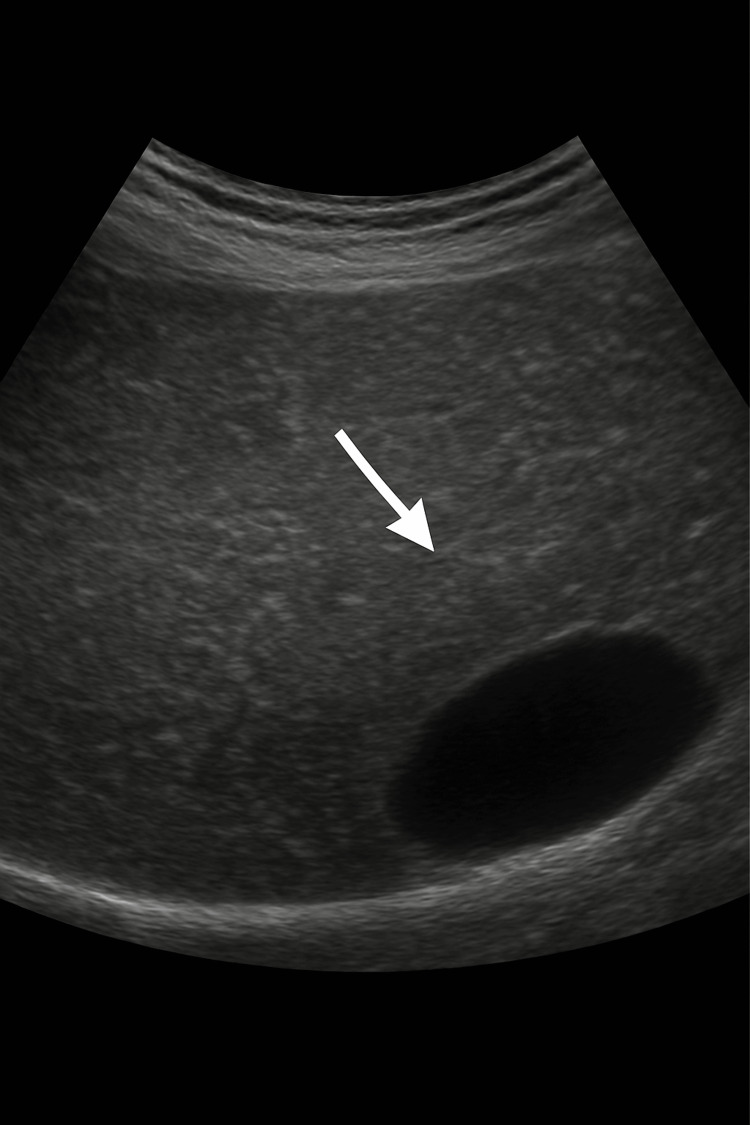
Abdominal ultrasound showing features of hepatic steatosis with increased echogenicity of the liver parenchyma

A 58-year-old man initially presented in 2018 with vague central abdominal discomfort and unintentional weight loss. His social history revealed chronic heavy alcohol consumption for several years, exceeding 70 units per week (~560 g of ethanol weekly). Biochemical evaluation demonstrated transaminitis with preserved synthetic liver function. Investigations excluded hereditary haemochromatosis (normal homeostatic iron regulator [HFE] genotype) and autoimmune hepatitis (negative antinuclear antibody [ANA], anti-smooth muscle antibody [ASMA], and liver kidney microsomal [LKM] antibodies). Abdominal imaging revealed hepatic steatosis, as seen in Figure 1. He was counselled regarding alcohol cessation and subsequently discharged from the hepatology service. Although he achieved a transient reduction in alcohol intake, sustained abstinence was not maintained.

Over the following years, he developed progressive symptoms of lethargy and fluid retention, including moderate ascites and peripheral oedema. He re-presented in late 2022 with worsening liver biochemistry, marked hypoalbuminaemia, and clinical signs of decompensated liver disease. His Child-Pugh score was C10. Hepatology reiterated the importance of alcohol abstinence and outlined the serious complications of cirrhosis, including variceal bleeding and death. He remained cognitively intact and maintained a healthy diet. He was referred to community alcohol support services, and an upper GI endoscopy was arranged.

**Figure 2:**
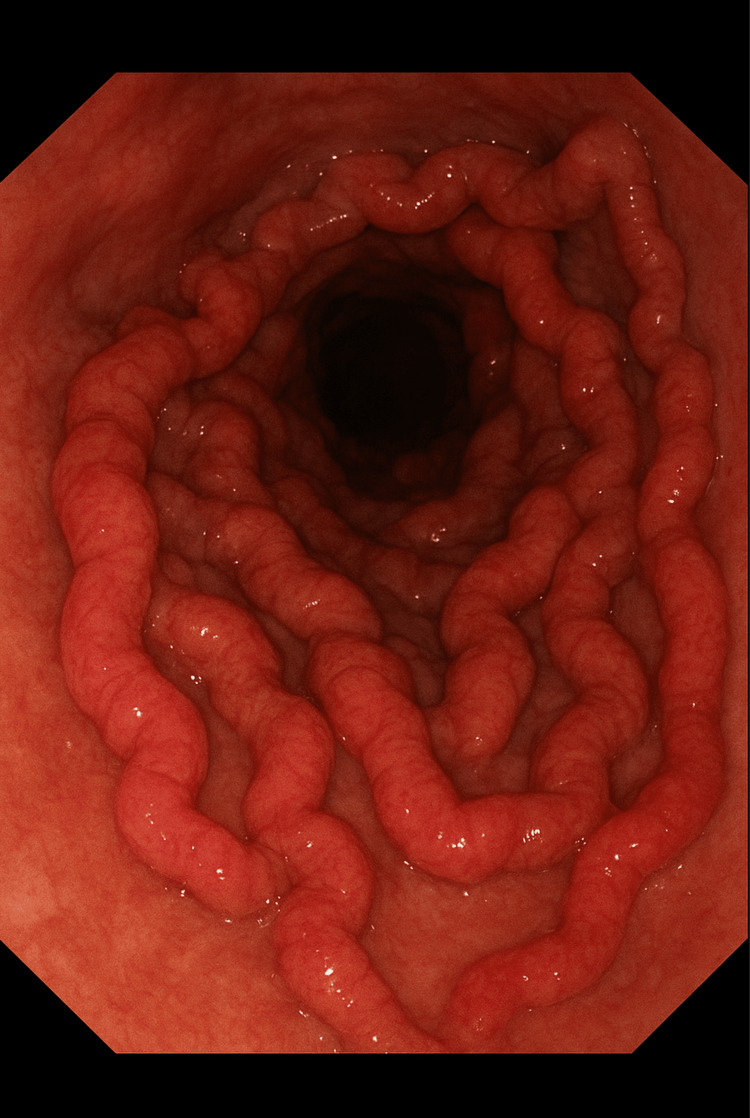
Oesophagogastroduodenoscopy (OGD) showing large oesophageal varices

Medical therapy was initiated, including spironolactone (100 mg once daily) and furosemide (20 mg once daily) for ascites, lactulose for hepatic encephalopathy prophylaxis, thiamine supplementation to prevent Wernicke’s encephalopathy, and carvedilol (3.125 mg twice daily) for variceal bleeding prophylaxis. The lower furosemide dose was chosen to maintain the recommended 100:20 mg spironolactone-to-furosemide ratio and prevent rapid fluid loss or electrolyte disturbance during initial diuretic titration. He was also found to have oesophageal varices requiring multiple oesophagogastroduodenoscopies, as seen in Figure 2, and, ultimately, Danis stent placement for uncontrolled variceal bleeding. During one of his admissions, he presented with spontaneous bacterial peritonitis, confirmed by positive *Escherichia coli* culture from ascitic fluid. Following successful treatment with piperacillin/tazobactam, he was commenced on long-term ciprofloxacin prophylaxis.

At this stage, he achieved sustained alcohol abstinence and was referred for liver transplant evaluation. During the initial transplant assessment in mid-2023, he was noted to have postural hypotension, hyponatraemia, and hyperkalaemia. He was admitted for inpatient management and initially treated conservatively, with transient improvement in blood pressure (systolic range 80-100 mmHg). However, biochemical abnormalities persisted, prompting referral to endocrinology for assessment of possible adrenal dysfunction while continuing the anti-liver failure regimen and changing PPI to an H₂ receptor blocker to minimise infection risk associated with long-term PPI use in cirrhosis.

A short Synacthen test, performed using 250 µg of synthetic adrenocorticotropic hormone (ACTH), tetracosactide, with serum cortisol measured at baseline and 30 minutes, revealed a low baseline cortisol of 170 nmol/L. The 30-minute cortisol value was unavailable, but the peak value was reported as below the normal reference threshold of 350 nmol/L, consistent with adrenal insufficiency. The post-Synacthen (delta) cortisol value was not available; however, the low baseline cortisol and inadequate rise on stimulation were consistent with adrenal insufficiency in the context of decompensated cirrhosis. ACTH levels were normal, and due to the clinical suspicion of relative adrenal insufficiency, salivary cortisol (free cortisol) was also requested for comparison, collected in the early morning (around 08:00) to correspond with peak physiological cortisol levels, though results were pending at the time.

Given his decompensated cirrhosis, persistent hypotension, and refractory hyponatraemia and hyperkalaemia despite adequate volume resuscitation, adrenal hypofunction was strongly suspected, and empirical corticosteroid therapy was initiated to prevent further haemodynamic compromise. Oral hydrocortisone (20 mg in the morning and 10 mg in the evening) and fludrocortisone 100 µg once daily were started. Routine blood tests were reviewed prior to initiating corticosteroids and repeated three weeks after starting treatment to assess biochemical response. Table 1 displays routine blood results before and after steroid initiation.

**Table 1:** Patient's laboratory test results.

	Values before starting steroids	Values after starting steroids	Normal range
Sodium	131 mmol/L	125 mmol/L	133 - 146 mmol/L
Potassium	4.5 mmol/L	5.0 mmol/L	3.5 - 5.3 mmol/L
Creatinine	78 µmol/L	93 µmol/L	Adult male: 59 - 104 μmol/L
Haemoglobin	85 g/L	94 g/L	Male: 130 - 170 g/L
Platelets	121 × 10^9^/L	103 × 10^9^/L	150 -400 x 10^9^/L
Albumin	23 g/L	25 g/L	35 - 50 g/L
Bilirubin	97 µmol/L	112 µmol/L	Adults: <21 µmol/L

At that point, sodium levels remained low, and potassium levels normalised as sodium zirconium cyclosilicate was introduced and spironolactone was discontinued. The rest of his medications were unchanged. As part of further investigations, he was also noted to have a markedly low total testosterone level of 3.22 nmol/L, consistent with hypogonadism likely secondary to critical illness and impaired hepatic production of SHBG and albumin. A summary of the full endocrine panel is given in Table 2. The sequence of events from transplant assessment to steroid initiation spanned approximately three weeks, during which serial assessments confirmed persistent hypotension despite supportive measures.

**Table 2:** Endocrine and biochemical laboratory values at initial assessment.

	Values	Normal range
Total serum cortisol/SST	170 nmol/L	≥ 350 nmol/L
Salivary cortisol	22.8 nmol/L	3 - 46 nmol/L
Adrenocorticotropic hormone	normal	5 – 50 ng/L
Luteinizing hormone	5.0 IU/L	Adult male: 1.2 – 8.6 IU/L
Follicle-stimulating hormone	11.9 IU/L	Adult male: 1.3 – 19.3 IU/L
Testosterone	3.22 nmol/L	Adult male: 8 - 30 nmol/L
Thyroid-stimulating hormone	3.85 mU/L	0.38 – 5.33 mU/L
Free T4	15.6 pmol/L	8.0 - 18 pmol/L
Prolactin	515 mU/L	Adult male: <550 mU/L

In the months following steroid initiation, his blood pressure remained persistently low with progressive clinical deterioration. Despite comprehensive medical management, including the above management plans, nutritional support with calorie-dense supplements, intensive care input for vasopressor support, and intravenous hydrocortisone, the patient remained refractory to maximal therapy and could not be optimised for liver transplantation. After multidisciplinary discussions involving his family and the hepatology, endocrinology, intensive care, and palliative care teams, a consensus decision was made to withdraw aggressive interventions. The patient died peacefully with his family at his bedside.

This presentation was consistent with adrenal hypofunction secondary to advanced cirrhosis, in which persistent inflammation, metabolic stress, and impaired hepatic synthesis blunt the adrenal stress response. Despite timely recognition and multidisciplinary intervention, his condition remained refractory, reflecting the complex interplay between endocrine and hepatic failure. Early suspicion for relative adrenal insufficiency in decompensated cirrhosis, coupled with careful interpretation of cortisol assays and proactive, team-based management, is essential to optimise outcomes in this vulnerable group.

## Discussion

Relative adrenal insufficiency in critical illness is a multifactorial condition characterised by a disruption in the hypothalamic-pituitary-adrenal axis and impaired adrenal responsiveness. The proposed pathophysiological mechanisms include systemic inflammation, endotoxaemia, and haemodynamic instability, which collectively suppress ACTH secretion, impair adrenal steroidogenesis, and reduce adrenal perfusion [8]. In decompensated cirrhosis, adrenal dysfunction is influenced by several disease-specific factors, including reduced cholesterol substrate, impaired cortisol biosynthesis, attenuated responsiveness to ACTH, and lower circulating levels of cortisol-binding proteins such as CBG and albumin. Some reports also suggest that altered tissue sensitivity to cortisol may contribute, although this remains less clearly defined [7].

These abnormalities may lead to biochemical profiles suggestive of adrenal insufficiency, particularly low total cortisol levels, despite preserved or even sufficient free cortisol concentrations. Consequently, adrenal dysfunction in cirrhosis is increasingly understood not as a fixed endocrine failure but as a context-dependent mismatch between cortisol supply and physiological demand [2,9].

This paradigm shift has significant implications for both the diagnosis and management of RAI in patients with advanced liver disease, where cirrhosis-related physiological alterations often confound standard markers of adrenal function. This evolving understanding reframes relative adrenal insufficiency as a dynamic and context-dependent phenomenon, carrying important implications for both its diagnosis and clinical management in this population [1,9].

Our patient demonstrated a low basal total cortisol level and an abnormal response to the short Synacthen test, initially supporting a presumptive diagnosis of relative adrenal insufficiency. However, a normal salivary cortisol level, which more accurately reflects free, biologically active cortisol, raised uncertainty regarding the true extent of adrenal impairment [9]. 

The presumptive diagnosis of relative adrenal insufficiency was based on low basal total cortisol and a suboptimal response to the standard-dose short Synacthen test (SD-SST). However, this approach is limited in cirrhotic patients due to reduced levels of cortisol-binding proteins such as albumin and corticosteroid-binding globulin, which can artifactually lower total cortisol concentrations and increase the risk of false-positive diagnoses [1,7].

Assessing adrenal reserve in patients with cirrhosis requires careful interpretation of several biochemical tests, each with its strengths and limitations. While total cortisol remains the most widely used screening tool, its interpretation must be contextualised within the unique biochemical milieu of liver disease. To address this, Wentworth et al. recommend evaluating the delta cortisol (i.e., the rise in cortisol following ACTH stimulation) rather than absolute values. A delta <250 nmol/L provisionally supports RAI [9]. In this case, the delta cortisol value was not recorded, representing a key diagnostic limitation that restricts direct comparison with standard interpretive criteria.

Free plasma cortisol (FPC), measured via equilibrium dialysis or liquid chromatography-tandem mass spectrometry (LC-MS/MS), and salivary cortisol are binding-independent biomarkers that more accurately reflect the biologically active fraction of circulating cortisol. Despite their theoretical advantages, their routine use in cirrhosis remains limited due to prolonged turnaround times, restricted availability, inter-laboratory variability, and the absence of validated diagnostic thresholds or established clinical utility in this population [9]. Similarly, calculated surrogates, including the free cortisol index, Coolens-derived free cortisol, and cortisol-to-CBG ratios, can be derived from routine biochemistry but perform inconsistently in patients with significant hypoalbuminemia. More complex tools, such as models of maximal cortisol secretion rate, require intensive serial sampling and are primarily restricted to research settings [9].

In light of these limitations, Wentworth et al. propose a pragmatic, tiered diagnostic algorithm. The initial evaluation should include an early-morning total cortisol measurement and a standard-dose short Synacthen test (SD-SST), with interpretation focused on the delta cortisol response. This diagnostic approach was applied to our patient. An early-morning total cortisol measurement followed by SD-SST demonstrated a suboptimal cortisol response, provisionally fulfilling criteria for RAI [9]. The salivary cortisol result (22.8 nmol/L) became available after steroid initiation, approximately three weeks later, at the time of endocrine reassessment, which further complicated interpretation. Although the patient was haemodynamically stable, he exhibited persistent electrolyte abnormalities and borderline hypotension, leading to the initiation of hydrocortisone and fludrocortisone therapy. Steroid therapy was commenced empirically during the same admission, and follow-up testing was performed three weeks later to evaluate biochemical and haemodynamic response.

Due to limited access to FPC and the absence of a recorded delta cortisol, a salivary cortisol measurement was performed instead, yielding a typical result (22.8 nmol/L). Nevertheless, glucocorticoid therapy was continued, given the ongoing diagnostic uncertainty. This sequence highlights the real-world challenge of managing RAI in cirrhosis, where clinical urgency often precedes confirmatory testing, and follow-up assessment may occur weeks later. In retrospect, although the recommended diagnostic pathway was followed, the clinical significance of the normal salivary cortisol was not adequately weighed. While Wentworth et al. acknowledge the theoretical utility of salivary cortisol in assessing adrenal function, they caution against its routine application in cirrhosis due to undefined reference intervals and assay variability [9]. However, in the context of clinical stability, a normal salivary cortisol may have reasonably argued against true adrenal insufficiency. It could have influenced the decision to withhold or de-escalate corticosteroid therapy.

The diagnostic assessment of RAI in cirrhosis is complicated by overlapping features between true endocrine dysfunction and the pharmacological effects of medications commonly used in advanced liver disease. In this case, the patient was receiving spironolactone, furosemide, carvedilol, lactulose, and long-term ciprofloxacin, all standard therapies in cirrhosis management. However, these agents can induce biochemical and haemodynamic changes that closely mimic the clinical profile of adrenal insufficiency, thereby complicating the interpretation of adrenal function tests. These medications had been introduced months before the endocrine work-up, during his period of clinical decline, suggesting that drug-related physiological effects may have compounded the apparent cortisol deficiency.

Spironolactone, for instance, can produce side effects such as hyponatraemia and hyperkalaemia [10], which overlap significantly with the clinical manifestations of both primary and secondary adrenal insufficiency [11]. When combined with furosemide, the risk of intravascular volume depletion and electrolyte derangements is further amplified, potentially leading to misleading biochemical findings. Importantly, while neither diuretic directly impairs cortisol synthesis, their systemic effects may simulate the phenotype of adrenal failure. 

Carvedilol, a non-selective beta-blocker with vasodilatory properties, has been shown in a meta-analysis to reduce mean arterial pressure in cirrhotic patients more than propranolol, alongside producing a greater fall in portal pressure [12]. While this haemodynamic effect supports its therapeutic use in portal hypertension, it could also complicate the clinical recognition of adrenal insufficiency, as hypotension and reduced perfusion are features standard to both conditions.

Furthermore, cirrhosis itself induces physiological alterations that can confound the diagnostic picture. Liamis et al. note that hyponatraemia in cirrhosis often arises from systemic arterial vasodilation and non-osmotic vasopressin release, hallmarks of the disease process [13]. Although hypotension is not explicitly discussed, it frequently coexists and may be exacerbated by the use of beta-blockers and diuretics. By the time of endocrine reassessment, these interacting factors - disease progression, medication effects, and possible receptor downregulation - collectively influenced the patient’s persistent hypotension.

In retrospect, the patient’s borderline hypotension and electrolyte abnormalities may have been more accurately attributed to the pharmacodynamic effects of these medications or to underlying cirrhotic pathophysiology, rather than to true adrenal insufficiency. This case underscores the need for a cautious, multidisciplinary approach when interpreting adrenal function tests in patients with cirrhosis, recognising that medication side effects may closely mimic endocrine dysfunction.

In this case, the initiation of empiric corticosteroid therapy was not associated with clinical improvement, as the patient continued to deteriorate despite medical optimisation. The absence of a haemodynamic response raises critical questions regarding the utility and potential harm of glucocorticoid treatment in cirrhosis when diagnostic certainty is lacking. This scenario underscores the importance of avoiding therapeutic inertia and highlights the necessity for ongoing reassessment of both diagnosis and treatment efficacy.

The use of corticosteroids in RAI remains controversial. Current evidence supporting routine glucocorticoid replacement in cirrhosis is limited, particularly in non-critically ill patients [14]. Wentworth and colleagues emphasise that decisions around steroid therapy in cirrhosis should be individualised. In critically ill patients with haemodynamic instability, a trial of glucocorticoids may be considered. From a management standpoint, clinicians should weigh the potential short-term haemodynamic benefits of steroid therapy against risks such as infection, hyperglycaemia, and muscle wasting [15]. In non-critically ill patients, however, the evidence base is limited, and routine empiric steroid therapy is not recommended. However, due to the uncertain long-term benefits and risks, any such intervention warrants close clinical monitoring and timely follow-up [9].

Moreover, prolonged exposure to exogenous glucocorticoids can paradoxically induce acquired glucocorticoid resistance. Wilkinson et al. note that long-term glucocorticoid exposure can reduce the availability of functional GRα. This occurs through several mechanisms, including suppression of gene transcription via nGREs, reduced stability of GRα mRNA, and accelerated receptor breakdown through phosphorylation-driven proteasomal pathways [16]. These molecular alterations have important clinical consequences, as they translate into reduced tissue responsiveness to glucocorticoids. Reduced glucocorticoid sensitivity can limit the effectiveness of steroid replacement, complicating the management of adrenal insufficiency [17]. In our patient, months of hydrocortisone and fludrocortisone therapy preceded further deterioration, a temporal association consistent with possible receptor desensitisation and declining steroid responsiveness.

Although it remains speculative whether discontinuation of glucocorticoids would have altered the outcome, particularly in the context of progressive multiorgan failure, the case reinforces a key principle: empiric corticosteroid therapy in cirrhosis should not proceed unchecked. Early and objective clinical or biochemical improvement should be evident; in their absence, clinicians must re-evaluate the underlying diagnosis, consider tapering or discontinuing therapy, and review the potential contributions of concurrent medications to ongoing electrolyte disturbances and hypotension. This iterative, cautious approach is essential to avoid both therapeutic futility and iatrogenic harm.

## Conclusions

This case exemplifies the diagnostic ambiguity and therapeutic challenges in managing presumed hepatoadrenal syndrome in the context of advanced cirrhosis. It underscores how cirrhosis-associated reductions in cortisol-binding proteins, hypoalbuminaemia, and the impact of frequently used medications (e.g., spironolactone, beta-blockers) can confound both the clinical presentation and interpretation of adrenal function tests. The case also highlights the limitations of relying solely on total serum cortisol levels, without accounting for altered protein binding or measuring free cortisol, particularly in critically ill patients, which is common in this context. Importantly, it illustrates the lack of consensus on diagnostic thresholds and the uncertain benefit of empiric corticosteroid therapy in this setting. In this case, persistent hypotension despite prolonged corticosteroid treatment, alongside the discrepancy between low total and normal salivary cortisol levels, reinforces the limited therapeutic benefit and diagnostic uncertainty of empiric steroid use in cirrhotic patients. Clinicians must therefore maintain a high index of suspicion while integrating clinical judgement, dynamic testing, and multidisciplinary input when evaluating adrenal dysfunction in end-stage liver disease.

Practical approaches to improve diagnostic accuracy include incorporating salivary cortisol or free cortisol measurement when feasible, reassessing the need for steroid therapy if there is no haemodynamic improvement, and engaging endocrinology and hepatology teams early in management. Future research should focus on developing clearer diagnostic criteria, validating the role of dynamic adrenal testing specific to cirrhosis, and identifying biomarkers that better predict steroid responsiveness. These advances would enable more targeted and safer management strategies for patients with suspected hepato-adrenal dysfunction.
